# The Oral Microbiota Changes in Orthodontic Patients and Effects on Oral Health: An Overview

**DOI:** 10.3390/jcm10040780

**Published:** 2021-02-16

**Authors:** Maria Contaldo, Alberta Lucchese, Carlo Lajolo, Cosimo Rupe, Dario Di Stasio, Antonio Romano, Massimo Petruzzi, Rosario Serpico

**Affiliations:** 1 Multidisciplinary Department of Medical-Surgical and Dental Specialties, University of Campania Luigi Vanvitelli, 80138 Naples, Italy; alberta.lucchese@unicampania.it (A.L.); dario.distasio@unicampania.it (D.D.S.); antonio.romano4@unicampania.it (A.R.); rosario.serpico@unicampania.it (R.S.); 2 Head and Neck Department, Fondazione Policlinico Universitario A. Gemelli IRCCS, Roma Università Cattolica del Sacro Cuore, 00168 Rome, Italy; carlo.lajolo@unicatt.it (C.L.); cosimo.rupe01@icatt.it (C.R.); 3 Interdisciplinary Department of Medicine, University of Bari “Aldo Moro”, 70121 Bari, Italy; massimo.petruzzi@uniba.it

**Keywords:** orthodontics, clear aligners, brackets, microbiota, microbiome, oral health, *Candida albicans*, periodontitis, caries, dental plaque, materials, PCR

## Abstract

Nowadays, there is a considerable interest to study the biological and microbiological changes that accompany orthodontic treatment. Growing knowledge on oral microbiota allows, day after day, to identify and characterize the microbial arrangements specifically associated with oral and extra-oral conditions. The aim of the present work is to highlight any further correlations between orthodontic appliances and the qualitative and quantitative modifications of the oral microbiota, such as predisposing factors for the onset of caries, periodontal diseases, and other infections, which can impact the oral and systemic health of the orthodontic patients. When compared with subjects without orthodontic appliances, orthodontic patients reported significant qualitative and quantitative differences in supra- and subgingival plaque during the entire treatment period. Certain components of fixed appliances (mainly bonded molar brackets, ceramic brackets, and elastomeric ligatures) showed high risks of periodontal disease and tooth decay for patients. An unclear prevalence of *Candida* spp. and the paucity of studies on viruses and protozoas in the oral microbiota of orthodontic patients need to be further investigated. The evidence emerging from this study could guide clinicians in modulating the timing of controls and enhance patient motivation to prevent the formation of mature plaque, thus reducing the risks of oral-plaque-related diseases.

## 1. Introduction: Oral Dysbiosis and Related Oral Conditions in General Population

Over the years, the need and prevalence of orthodontic treatments have increased, particularly in developed countries [1], for both therapeutic and aesthetic purposes, and thanks to the development of different orthodontic devices and protocols [2,3,4,5] that meet the needs of every potential patient. Orthodontic procedures are indicated to correct dental malocclusions and craniofacial skeletal discrepancies of a genetic, family, and/or environmental nature, using fixed and/or removable orthodontic devices.

Recently, research has also investigated various biomarkers to monitor biological changes in tooth movement before and during treatment and has focused on the role and weight of numerous variables, such as oral hygiene levels and food habits related to the onset of dental and periodontal diseases during orthodontic treatment [6,7].

In general, the main plaque-related periodontal diseases are gingivitis and periodontitis. Gingivitis is a non-destructive inflammation, usually reversible after dental plaque control, while periodontitis, determined by the presence of predisposing factors—genetic and environmental—continues even after the restoration of oral hygiene [8], resulting in the irreversible loss of attachment and teeth due to a perpetuation of local inflammation, initially triggered by periodontopathogen bacteria [9]. 

In addition, various systematic reviews have confirmed the worsening of clinical parameters indicative of periodontal diseases, such as plaque index, bleeding on probing, attachment loss, and the onset of pockets or gingival recessions, in association with the time and type of orthodontic treatment, reporting varying degrees of reversibility after treatments.

The causes of the onset of periodontal diseases in orthodontic patients can be traced to:

- more difficult maintenance of oral hygiene,
- plaque retention to orthodontic devices,
- bone/periodontal movements and remodeling under orthodontic forces, which can favor the accumulation of subgingival plaque and enhance the periodontal pathogenic potential [10,11].

Aside from periodontal implications, plaque retention in the presence of orthodontic appliances has also been associated with increased susceptibility to caries and is influenced by transient/permanent qualitative and quantitative changes in the oral microbiota, responsible for the onset of oral infections, with systemic effects on well-being [12,13], thus predisposing orthodontic patients to suffer various pathologies more likely than non-orthodontic patients [14].

### 1.1. Dental Plaque Characterization 

Dental plaque changes over time and depending on the location, so that early and mature plaques, supragingival and subgingival plaques, differ greatly from each other and are responsible for various and different pathologies, the most prevalent of which are caries and periodontal diseases. This is due to the fact that, by definition, dental plaque is a polymicrobial biofilm made up of various bacterial complexes, which mutually benefit from coaggregation, adhesion, and metabolic interactions [15].

Supragingival plaque is generally considered to be the main cause of caries and demineralization of enamel and dentin, as a consequence of the presence of *Streptococcus mutans* and other cariogenic bacteria, such as *Lactobacilli* and *Actinomycetes* species (spp.) as second colonizers, [14] but it also plays a key role in the late co-aggregation of periodontopathogen bacteria in the subgingival plaque, consisting mainly of gram-negative anaerobic bacteria strongly associated with periodontal diseases [16].

Thanks to culture-independent high-throughput technologies, such as reverse transcriptase-polymerase chain reaction *(*RT-PCR) [17], and related, which, compared to conventional culture methods widely used in the past, allow the simultaneous evaluation of a large number of microbial species from different samples—saliva, supra-, and sub-gingival plaque—and from a wide range of subjects, the knowledge on the oral microbiota is enriched, day by day, allowing the characterization of the microbiota in conditions other than baseline. By definition, the oral microbiota is the collection of more than 600 microbial species—eukaryotes, archaea, bacteria, fungi, and viruses—living in specific ecological niches of the mouth [18,19], 169 of which constitute the indigenous “core oral microbiome”, whose species are qualitatively and quantitatively housed in three different niches, classified as: Group 1, buccal mucosa, keratinized gingiva, and hard palate; Group 2, saliva, tongue, tonsils, and throat (back wall of oropharynx); and Group 3, sub- and supra-gingival plaque [20].

#### 1.1.1. Subgingival Plaque and Periodontopathogen Bacteria

Subgingival plaque was first characterized in 1998, with checkerboard DNA–DNA hybridization (CDDH) when Socransky et al. analyzed the subgingival microbiota in a series of adults with and without periodontitis and grouped the forty species more frequently associated into complexes [21]. The grouping of these complexes was done on the basis of their strong or weak association with periodontitis and its clinical signs and they have been color-labeled in red, orange, yellow, green, and purple complexes; even today, this classification is used to refer to the periodontal pathogenic potential of each specific bacterium.

Periodontal damage begins thanks to the so-called “early colonizers” (the bacteria of the green and yellow complexes), which are able to adhere with their fimbriae to the dental film, thus favoring the subsequent co-adhesion and co-aggregation of the bacteria of the orange complex. The orange bacteria are the “bridge species” that connect green and red bacteria, produce toxins and enzymes responsible for the progressive loss of attachment and increase in pocket depth, thus creating a hospitable environment in the gingival sulcus/pocket for living conditions and colonization by red-complex bacteria. The latter is the “late colonizers”, lodged in the deepest pockets and strongly associated with bleeding in the advanced stages of periodontitis [21].

Therefore, periodontal damage by red bacteria is the endpoint of a process during which different green/yellow and orange bacteria accumulate and co-aggregate, making the subgingival niche a hospitable habitat for the red bacteria.

#### 1.1.2. Supragingival Plaque and Periodontopathogen Bacteria

Ten years later of their first work, Haffajee, Socransky et al. repeated a similar investigation to define relationships among bacterial species in supragingival plaque samples from a control group of periodontally healthy subjects and a test group consisting of patients who experienced attachment loss [22]. They found specific microbial complexes in the test group’s supragingival plaque that were similar to those found in subgingival plaque samples, with some minor differences. Moreover, in this case, the colonization sequence of the supragingival plaque begins with the *Streptococci* (yellow complex), which are progressively replaced by the *Actinomicetes,* and then, at the bleeding sites and in the gingival pockets, they bind to the orange and red complexes, both ubiquitously present in supragingival and subgingival plaques and in inflamed and non-inflamed pockets.

### 1.2. The Role of Other Microscopic Agents in Gingival and Oral Health

Noteworthy, virus, fungi, and protozoa may also be involved in gingival diseases, such as linear gingival erythema associated with *Candida albicans* in children with AIDS [23] or *Entamoeba gingivalis* involved in periodontal destruction [24].

As regards *Candida* spp., they are not only responsible for a series of oral and systemic opportunistic infections [25,26], but they have also been implicated in the onset of caries as they show a synergistic relationship with *S. mutans*, with which they are frequently associated in mature plaque, mainly in children [27].

Usually, the presence of viruses in the oral microbiota (the so-called “oral virobiome”) is indicative of potential infections, as the viruses are microscopic agents responsible for infection and damage to eukaryotic cells, with lifelong effects and/or recurrences [28,29]. Among the viruses commonly found at the oral sites, Epstein–Barr Virus (EBV) and Human Cytomegalovirus (CMV), as well Herpes Simplex Virus (HSV), also showed significantly higher in patients with severe periodontitis than in healthy subjects [30,31] and viral–bacterial associations in aggressive periodontitis—such as EBV/CMV with *Porphyromonas gingivalis* and *Aggregatibacter actinomycetemcomitans*—has been reported by various authors [32,33]. These viruses have been considered responsible for local immunosuppression that can favor the subgingival colonization and the multiplication of periodontal bacteria, indirectly exerting a periodontopathic effect [32,33].

### 1.3. Aims

On the basis of the evidence reported for the general population, the aim of the present work is to highlight any further correlations between orthodontic appliances and the related qualitative and quantitative modifications of the oral microbiota, such as predisposing factors for the onset of caries, periodontal diseases and other infections, which can impact the oral and systemic health of the orthodontic patients.

## 2. Microbiota Changes in Orthodontic Patients with Fixed Appliances

Several systematic reviews summarized the clinical and microbial differences found in salivary or plaque samples between orthodontic patients (case groups) and subjects not undergoing orthodontic therapies (control groups).

Pan et al. found significant differences in the number of microbial counts between 61 orthodontic and 56 non-orthodontic subjects (age range, 11–17 years) [34], reporting the highest increased in the case group three months after the placement of the orthodontic appliances and the significantly increased presence of strongly pathogenic *P. gingivalis-specific fimA* genotypes in the case group, defining this subtype as closely associated with orthodontic gingivitis. 

The consistent quantitative and qualitative changes in the plaque one month after the start of orthodontic treatment were confirmed by a systematic review by Lucchese et al. [11]. According to it, different orthodontic appliances tended indiscriminately to alter the oral microbiota during treatment, but fixed appliances reported a greater and significant cariogenic and periodontopathogen effect than removable appliances.

### 2.1. Fixed Appliances and Periodontopathogen Bacteria 

In 2017, Guo et al. [35] performed a meta-analysis to observe microbial changes in subgingival plaque between case/controls and before, during, and after orthodontic treatments with metal fixed appliances, focusing on the amounts of the red-complex bacteria (*P. gingivalis, Prevotella intermedia* and *Tannerella forsythia*, plus *A. actinomycetemcomitans)***.** Of these, only *T. forsythia* showed a statistically significant increase three months after orthodontic appliances, while a transient increase in all the species considered was detected six months after the start of the treatment. Furthermore, *P. intermedia* characteristically increased more significantly at the incisors than at the molars. 

In 2018, Sun et al. [36] investigated microbial diversity from salivary samples from 20 healthy individuals and 30 patients with full fixed orthodontic appliances during the midterm of orthodontic treatment (10–12 months): real-time PCR analysis with gene-specific primers confirmed that the two groups expressed two distinct microbial communities with greater microbial diversity in the orthodontic group, characterized by higher amounts of *Pseudomonas* spp., *P. synxantha, Burkholderia species* and *Veillonella parvula*; conversely, there were no significant differences in *S. oralis* and *Neisseria lactamica* amounts.

The association between changes in clinical parameters and subgingival plaque was highlighted by Naranjo et al. in cultures from subgingival microbial specimens collected from 30 patients before and after bracket placement and 30 additional control subjects without orthodontic treatment [37]. Compared to the control group and baseline, elevated levels of *P. gingivalis, P. intermedia/Prevotella nigrescens, T. forsythia,* and *Fusobacterium* spp., were found in orthodontic patients three months after the placement of the brackets. Furthermore, a number of gram-negative superinfecting bacteria and enteric rods (*Klebsiella oxytoca, Enterobacter cloacae, Klebsiella pneumoniae, Serratia marcescens, Pseudomonas species, Enterobacter aerogenes, Enterobacter gergoviae*) were found indistinctly in all groups, without significant differences. Similarly, Kim et al. studied longitudinal changes in the subgingival microbiota by PCR before and during the first period of orthodontic treatment (up to 6 months) [38] in 30 adolescents treated with fixed appliances (conventional metal brackets with stainless steel ligatures and molar bands). From three months after placement of orthodontic appliances and thereafter, at least one periodontopathogen species—among *A. actinomycetemcomitans, T. forsythia, C. rectus, Eikenella corrodens, P. gingivalis, P. intermedia, P. nigrescens, and Treponema denticola—*was found in every subject. Among them, *C. rectus and P. nigrescens* significantly increased already after the first week of treatment, while *T. forsythia* took longer to colonize, after 3–6 months of treatment. In contrast, *A. actinomycetemcomitans* was detected at a very low level and showed no significant increase during the observation period. All frequencies of periodontopathogens were significantly higher in the molars than in the incisors. A similar decrease in *Actinomyces* spp. after 1 year of treatment was also reported by Lemos et al. in a study with CDDH on subgingival biofilm samples from 17 adults with full-fixed orthodontic appliances [39]. These authors also reported that, during treatment, *P. intermedia* and other orange complex species increased, while the proportions of the red complex species remained unchanged.

In addition to considering microbial changes *during* therapy, other authors have focused on changes that happen *after* the end of therapy, to assess whether a return to baseline occurs or if an altered microbiota remains. In their work, Guo et al. also reported a return to pretreatments levels several months after the removal of fixed appliances [35]. This was also confirmed by Choi et al. with a PCR-based study of the microbial changes occurring in subgingival plaque samples from 30 orthodontic patients three months after removal of fixed orthodontic appliances compared to 30 healthy subjects not undergoing orthodontic treatment [40]. They also reported that periodontopathogens bacteria significantly increased during orthodontic treatment (mainly *T. forsythia, C. rectus, and E. corrodens*) and then significantly reduced within three months of removing the appliance, eventually returning to levels similar to those reported in healthy subjects, except for *T. forsythia* and *P. nigrescens,* which persisted at even higher but not statistically significant levels compared to the control group. Similarly, Lo et al. [41] described with cultural techniques the bacterial composition before and during fixed orthodontic treatment in 10 patients (age range, 10–17 years), up to 12 weeks from appliance placement, revealing a prevalent aerobic flora (*Streptococcus* spp., *A.odontolyticus, A. israelii*) before treatment and one year from the start, and a prevalent facultative aerobic (*A. actinomycetemcomitans, Actinomyces viscosus, Capnocytophaga gingivalis)* and anaerobic species *(E.corrodens, Fusobacterium nucleatum, Micrococcus micros, Peptostreptococcus anaerobius, P. gingivalis, and Pseudomonas* spp.) during the first 2–4 weeks of treatment.

### 2.2. Fixed Appliances and Cariogenic Species

In 2016, Klaus et al. reported the results of a cross-sectional study on 75 subjects treated with fixed appliances (mean age, 14.4 ± 1.8 years) to measure the prevalence in saliva and plaque of *Candida spp, S. mutans, and Lactobacilli,* with culture methods [42]. The subjects were divided according to their oral hygiene status into three groups: good oral hygiene (GOH), poor oral hygiene (POH), and poor oral hygiene with white spots lesions (POH/WL). All patients, regardless of groups, reported a high prevalence of *Candida spp*. both in saliva (73% of cases) and plaque (61% of cases) with a significantly higher prevalence in the POH group and in the POH/WL group compared to the GOH group. *C. albicans* and *C. dubliniensis* were the main species identified, found in 86% and 15% of cases, respectively. With regards to bacteria, both *S. mutans* and *Lactobacilli* were found in 100% of salivary samples and in 91% of plaque samples, respectively, higher and significantly higher, in POH and POH/WL groups than in GOH group. A similar study on the cultural identification of salivary *S. mutans, Lactobacillus spp. and C. albicans* was performed by Topaloglu-Ak et al. [43] on 35 children with fixed appliances and 34 with removable devices, reporting a significant increase in *S. mutans* and L*actobacilli* six months after insertion of fixed/removable appliances and a statistically higher presence of *C. albicans* in the fixed appliance group than in the removable appliance group.

In 2011, Andrucioli et al. [44] assessed bacterial contaminations after 30 days of permanence of premolar bands inserted after 16 months of fixed orthodontic treatment in 18 patients (age range 11–29 years), by checkerboard DNA–DNA hybridization. Among the cariogenic species, *S. mutans and Streptococcus sobrinus* were found in greater numbers than *Lactobacillus acidophilus* and *Lactobacillus casei*. The detection of periodontal pathogens of the orange complex was higher than that of the red complex and represented 40% of the total bacterial count.

### 2.3. Conventional Brackets with Elastomeric Ligatures, Steel Ligatures, Self-Ligating Brackets and Ceramic Brackets

Among fixed appliances, metal brackets with elastomeric ligatures have been shown to retain more plaque and worsen bleeding on probing and the plaque index more significantly than steel ligatures. In detail, Türkkahraman et al., after performing a split-mouth study on 21 subjects with two different archwire ligation techniques (elastomeric rings and steel ligatures), reported more bleeding at the teeth ligated with elastomeric rings than those with steel ligatures [45]. This finding was confirmed by Alves de Souza et al. which reported significantly higher amounts of *T. forsythia* and *P. nigrescens* at elastomeric ligatures, while *P. gingivalis*, *A. actinomycetemcomitans* and P*. intermedia* did not differ significantly [46].

A third type of ligature used for archwire ligation is the self-ligating bracket: this system has been variously associated with higher bleeding, worse plaque index and with an increase in gram-negative and gram-positive bacteria (mainly *Streptococci and Lactobacilli*) in several studies, despite they did not report statistically significant differences compared to conventional brackets ligated with stainless steel ligatures [47,48]. Regarding the risk of caries, Jing et al. [49] found a significant increase in *S. mutans* in patients with conventional brackets compared to those with self-ligating brackets over 18 months after starting the treatments.

In 2002, Anhoury et al. [50] compared the total bacterial counts and the levels of various cariogenic and periodontopathogen bacteria present on 24 metallic and 32 ceramic orthodontic brackets immediately after their debonding. The mean counts of *P. gingivalis* were very similar on metallic and ceramic brackets isolated from both posterior and anterior teeth, as well for amounts of *P. nigrescens*, *Actinomyces odontolyticus*, *T. forsythia*, *Actinomyces naeslundii,* *Capnocytophaga ochracea, Actinomyces israelii* and cariogenic bacteria such as *S. mutans and L. acidophilus*. In contrast, the two types of bracket exhibited statistical differences in the counts of eight periodontopathogen species: the metallic bracket showed significant increased levels of *T. denticola, A. actinomycetemcomitans, F. nucleatum ss vincentii, S. anginosus* and *E. nodatum*, while in ceramic ones *E. corrodens,* *Capnocytophaga showae, Selenomonas noxia* were the most significantly increased species. Counts of *Streptococcus sanguis, Actinomyces gerencseriae, and Streptococcus constellatus* significantly increased in anterior ceramic brackets, while *C. rectus* was higher in posterior metallic brackets.

### 2.4. Molar Band vs. Bonded Molar Tubes

In 2014, Ireland et al. [51] used denaturing gradient gel electrophoresis (DGGE) and 16S rDNA microarray in a cross-mouth study on 24 orthodontic patients (age range, 11–14 years) to investigate differences in clinical parameters and microbial communities in supra- and subgingival plaque from banded molar vs bonded molar, randomly assigned, during treatment and up to one year after appliance removal. In both groups, the plaque populations changed within three months of starting fixed treatment and was characterized by an increase in *T. denticola* and *P. nigrescens*, and a decrease in *A. actinomycetemcomitans*. Post-treatment plaque associated with both types of molar attachments showed increased levels of periodontal pathogens, such as *P. gingivalis, T. forsythia*, and *E. nodatum*, while *C. rectus, Parvimonas micra, A. odontolyticus* and *V. parvula* were peculiarly elevated only in bonded molars. One year after the cessation of treatment, the banded molar plaque returned to its baseline composition, while a new arrangement of the microbial community persisted in the bonded molars.

In 2016, Mártha et al. [52] used DNA-strip technique to assess the presence of eleven periodontopathogen bacteria in subgingival plaque of banded and bonded molars, before and during the first two months of fixed orthodontic treatment, in 25 subjects (age range, 11–17 years). The most common bacteria, regardless of groups and time, were *F. nucleatum* (92% of samples), *E. corrodens* (76% of samples), and *Capnocytophaga* spp. (*C. gingivalis, C. ochracea, C. sputigena*), while the other eight species (*A. actinomycetemcomitans, P. gingivalis, P. intermedia, T. forsythia, T. denticola, Parvimonas micra, C. rectus, and Eubacterium nodatum*) were found less frequently. Among them, *E. nodatum* was found only in 2 subjects with bonded molars during treatment, while *E. corrodens, P. micra, T. denticola and T. forsythia* were stable over time in the banded molar group but it significantly increased two months later in the bonded group, as well as *Capnocytophaga spp*.

## 3. Microbiota Changes in Children with Functional/Orthodontic Appliances 

Various functional/orthodontic appliances are used in interceptive orthodontics, such as in those children needing rapid maxillary expansion. The microbial changes associated to these types of appliances were studied by Ortu et al. [53] who evaluated the microbial levels of *S. mutans* and *Lactobacillus* spp. in 30 children aged 6–9 years and grouped into 10 subjects treated with rapid palatal expander (RPE), 10 treated with Mc Namara expander, and 10 patients as control. These authors found a significant increase in *Streptococci and Lactobacilli* in each group but no significant differences between the groups, except the Mc Namara group that showed significantly higher amounts of *Lactobacilli* after 6 months of treatment than in control group and RPE. 

## 4. Clear Aligners vs. Fixed Orthodontic Appliances in Adults 

In 2015, Levrini et al. [54] prospectively considered 77 patients grouped in subjects with clear aligners, fixed orthodontics, and control to assess periodontal indexes and microbial composition of the plaque before and during treatments: they found better control over biofilm formations and significantly less severe periodontal indexes in the clear aligners wearers, as confirmed by the meta-analysis made by Rossini et al. on the periodontal health of patients treated with clear aligners compared to those treated with fixed appliances [55].

With regard to the characterization of the microbiota in subjects with clear aligner vs. fixed appliances, Lombardo et al. [56] recently reported a longitudinal study of the subgingival microbiota in these two groups, focusing on the identification and quantification of *A. actinomycetemcomitans, P. gingivalis, F. nucleatum, C. rectus, T. denticola* and *T. forsythia* by real-time PCR. They found a significantly increased total bacterial load during both treatments and a progressive increase in *C. rectus* and *F. nucleatum* in the fixed appliances group. Conversely, *A. actinomycetemcomitans* was never detected, while P*. gingivalis, T. forsythia* and *T. denticola* were rarely detected. Similar comparisons were also reported by Mummolo et al. [57], focusing on the amounts of *Streptococci and Lactobacilli* in a prospective controlled study on 80 adults—40 subjects with clear aligners and 40 with fixed orthodontic appliances—reporting a significant increase in the amounts of those cariogenic species in the saliva of subjects with fixed appliances.

The superior periodontal health in removable appliances wearers compared to fixed orthodontic appliances has recently been confirmed by two meta-analyses by Lu et al. and Wu et al. [58,59]. The authors considered randomized controlled trials (RCTs) and prospective cohort studies comparing periodontal health status in patients undergoing orthodontic treatment with fixed and removable appliances. They compared the gingival index (GI), plaque index (PI), the sulcus probing depth (SPD), and the sulcus bleeding index (SBI) from seven and thirteen eligible studies, respectively, and found that, compared to wearers of fixed appliances, wearers of clear aligners did not show significant differences in GI and SPD but reported significantly lower PI and SBI, at any time of observation.

## 5. Protozoas, Fungi and Other Bacterial Species in Orthodontic Patients

While a wide variety of works have focused on cariogenic and periodontopathogen bacteria in orthodontic patients, further studies focused on other pathogenic and opportunistic microorganisms responsible for various oral and systemic diseases. In 2019, Perkowski et al. performed a cultural study on periodontal swab from 25 young orthodontic patients (6–13 years old) treated with removable orthodontic appliances and 25 older patients (14–23 years) treated with fixed appliances, each compared to 50 healthy subjects in the same age ranges of the two test groups [60] to test for any clinical and microbial differences among the four groups. As expected, caries and DMFT (decay, missed, filled teeth index) [61] were significantly higher in the younger subjects, while bleeding on probing was higher in the older subjects, irrespective of case/control groups. The fixed orthodontic group showed a higher prevalence of *Enterococcus faecalis, Enterococcus faecium, Staphylococcus aureus and Escherichia coli*, and other gram-negative such as *Enterobacter cloacae, P. agglomerans, and Klebsiella* spp. plus various strains of *Candida spp*., compared to those with removable appliance or not treated. In particular, the presence of *C.albicans* was correlated with higher bleeding indices and poor oral hygiene. Furhtermore, few older subjects reported the identification of protozoas (*Trichomonas tenax* and *Entamoeba gingivalis)* regardless of the groups, while cysts of *Acanthamoeba spp*. were exclusively found in 3 patients with fixed appliances.

The higher carriage of *Candida spp*. in orthodontic patients with fixed appliance than in non-orthodontic subjects and patients with orthodontic removable appliances has been previously discussed, as reported by Topaloglu et al. [43] and other authors [62,63]. Klaus et al. also reported a significant higher prevalence of *Candida spp*. associated with poor oral hygiene [42], as confirmed by the literature [64].

In their work, Arslan et al. characterized by culture methods *Candida* species found in 58% of a group of adolescents with fixed orthodontic appliances: *C. albicans* was predominant (73.8%), followed by *C. tropicalis, C. krusei* and *C. kefyr* (7.14%), and *C.parapsilosis* (4.76%) [63]. Similar rates were reported by Grzegocka et al. [65] who analyzed *Candida spp*. from oral rinses and suprangingival plaque in 17 orthodontic subjects (mean age 17 ± 7 years) founding 59% of Candida carriers among them, and a strongly correlation with the massive presence of plaque.

Contrasted with these findings, Sanz-Orrio-Soler et al. [66] recently reported the results of a prospective controlled trial on oral colonization by *Candida* species in 124 orthodontic patients before, during, and after treatment with fixed appliances. Again, *Candida spp*. identification was based on culture methods and results contrasted with the previously published literature: the authors did not observe any statistically significant increase in the colonization of *C. albicans* during the orthodontic treatment despite the fact that ceramic brackets showed a higher incidence of this fungus. Similarly, Tapia et al. [67] found colonization rates of *C. albicans* and *C. tropicalis* of 6.7% in 90 orthodontic subjects (20.6 ± 7.1 years).

## 6. Discussion

The present work examined the scientific literature to highlight any specific microbial changes that occur during and after different types of orthodontic treatments.

When compared with subjects without orthodontic treatment, orthodontic patients reported significant qualitative and quantitative differences in the amount and microbial composition of plaque during the entire treatment period. Removable appliances were less associated with worsening of periodontal indices and caries because, despite being worn nearly 24 h a day, they can be easily removed to allow for proper oral hygiene. These results are in line with a recent meta-analysis by Jiang et al. [68], reporting that patients with clear aligners, compared with those undergoing orthodontic treatment with fixed appliances, appear to benefit from better oral health and periodontal parameters, thus recommending clear aligners as preferential therapeutic option in those patients at high risk of developing gingivitis/periodontitis, mainly adults [69]. Furthermore, subjects with fixed appliances showed a significant increase in the number of *Streptococci* and *Lactobacilli* and, therefore, a greater risk of caries than subjects with clear aligners [57].

Among the types of brackets and ligatures used in fixed orthodontic therapies, patients with conventional brackets have shown an increase in *S. mutans* and should be considered at higher risk of developing white spot lesions and caries than patients with self-ligating brackets [49], as well as elastomeric ligatures and ceramic brackets are more associated with poor oral conditions and with a greater amount of periodontopathogen and cariogenic species. Based on these findings, while self‑ligating brackets are microbiologically safer, elastomeric ligatures and ceramic brackets must be considered at higher risk of periodontal diseases and caries.

In fixed appliances, the first changes already occur during the first weeks of treatment and, within the first month, *Streptococci* and *Actynomicetes* spp. predominate, as well as *C. rectus* and *P. nigrescens* (orange complex species), *A. actinomycetemcomitans*, A*. viscosus, Capnocytophaga gingivalis* and anaerobic species (*Eikenella corrodens, F. nucleatum, Micrococcus micros, Peptostreptococcus anaerobius, P. gingivalis and Pseudomonas species*). After 3 months, the species of the orange complex increase and account for the 40% of the total bacteria counts. The red complex bacteria *P. gingivalis, P. intermedia,* and *T. forsythia*, significantly increase after 6 months, as well as *Lactobacilli*. From the end of the treatment, the literature agreed in founding a return to the original bacterial flora of the baseline, thus considering all the microbial alterations occurred during orthodontic treatment as transitory [35]. However, some authors have disproved this finding by reporting a long-term effect on periodontal health in patient with molar bonded tubes appliances compared with the classical molar bands [51]. On this basis, more rigorous follow-up should be considered after orthodontic treatment in patients treated with molar bond.

Regarding the presence of *Candida* spp. in orthodontic patients, many studies reported a high and significant prevalence of this yeast in orthodontic patients, but other studies have refuted this hypothesis. An explanation of these discordant results can be given by analyzing the reported studies: few subjects were enrolled and the methods of fungal detection were based on culture, which underestimate the presence of those yeasts that are difficult to cultivate, especially if present in low percentages. For these reasons, more extensive studies are desirable and the use of high throughput technologies should be preferred to culture methods in order to map, without risk of underestimation, the real oral mycobiome of orthodontic patients. It should be noted that *Candida* is an opportunist agent which, under physiological conditions, is present only as a carrier. However, if local or systemic alterations occur, it can virulent with repercussions of various degrees on the health of the individual [26].

In one study, the presence of protozoas was also found: *Trichomonas tenax*, *Entamoeba gingivalis,* and *Acanthamoeba spp*. are not a common resident of the oral cavity and their opportunistic colonization may be associated with serious systemic conditions, such as lung abscesses, granulomatous encephalitis, and keratitis, as well their proteinases activity is capable of damaging oral mucosal tissues and invading the periodontal spaces [58].

With regard to virobiome in orthodontic subjects, the scientific literature is silent and has not reported significant works on the presence of viruses associated with orthodontic treatments. However, based on the viral–bacterial interaction found in periodontitis [32], it is reasonable to hypothesize a triggering role of HSV, EBV, and CMV (viruses frequently reported in children and adolescents [70,71]) in periodontal damage if associated with significant amounts of periodontopathogen bacteria.

## 7. Conclusions

The qualitative and quantitative changes in the microbial plaque of orthodontic patients occur as early as one week and they become more consistent three months after the start of treatment, with stable colonization first by orange and then by red species. Especially in patients being treated with fixed devices, it is advisable to strengthen oral hygiene and clinical checks in the first months of treatment to block the progression, maturation, and arrangement of periodontopathogen and cariogenic species in plaque.

The results of the present review were in accordance with what recently reported by Müller et al., which concluded the reversibility of the periodontal changes and the permanence of enamel demineralization and white spots, thus suggesting the use of antibacterial orthodontic bonding systems as an adjunct in the maintenance of proper dental health in orthodontic patients [72].

Furthermore, this study focused on the literature related to healthy, immunocompetent subjects without other local or systemic affections. Therefore, it is foreseeable that patients with special needs, those with immunological alterations or other oral and/or systemic comorbidities, may be more affected by the cariogenic and periodontopathogen effects of mature plaque during orthodontic treatments, and a high carriage of *Candida* spp. could be responsible for fungal stomatitis and disseminated candidiasis in fragile subjects.

Since the correlations between oral dysbiosis and systemic diseases are various and reported in the more recent literature, [73,74] it is desirable to conduct targeted research on the less investigated components of the oral microbiota (fungi, viruses, and protozoan) and on fragile populations requiring orthodontic treatments, to prevent oral and extraoral complications and to guarantee each patient personalized dentistry with personalized therapies [75], which takes into account the microbiological and clinical characteristics of each individual.
